# Clinical validation of a high‐definition mid‐position magnetic resonance imaging approach for lung radiotherapy planning

**DOI:** 10.1002/mp.70457

**Published:** 2026-05-05

**Authors:** Katrinus Keijnemans, Astrid L. H. M. W. van Lier, Pim T. S. Borman, Hilâl Tekatli, Jacquelien Pomp, Romy E. van Weelderen, Bas W. Raaymakers, Martin F. Fast

**Affiliations:** ^1^ Department of Radiotherapy University Medical Center Utrecht Utrecht The Netherlands

**Keywords:** 4D‐MRI, mid‐position, lung cancer

## Abstract

**Background:**

Respiratory‐correlated four‐dimensional (4D) magnetic resonance imaging (4D‐MRI) is useful to estimate breathing induced motion for MRI‐guided radiotherapy. Based on 4D‐MR image sets, a three‐dimensional mid‐position (MidP) MRI can be generated using deformable image registration (DIR) for radiotherapy planning. However, the desired spatial resolution and image contrast of the MidP MRI may differ from the original 4D‐MRI.

**Purpose:**

This retrospective study validates a high‐definition (HD)‐MidP MRI approach that combines 4D‐MRI motion information with a high‐resolution MRI to enhance the spatial resolution of the MidP image.

**Methods:**

Computed tomography (CT) and MR image sets of 25 lung cancer patients were eligible, of whom 17 were complete and suitable for analysis. Standard‐definition (SD)‐MidP images were derived by applying DIR to warp the ten respiratory phases of a 4D‐CT or 4D‐MRI, whereas the HD‐MidP MRI was derived by warping a high‐resolution respiratory‐triggered MRI to the MidP. The MidP image quality was assessed with a 4‐point Likert scale on tumor and organ at risk (OAR) distinctiveness by three readers. Additionally, the gross tumor volume (GTV) was delineated by the readers, from which a consensus contour was derived for each MidP image. Reader contours were evaluated using the Dice similarity coefficient (DSC) and mean distance to agreement (DTA). Anatomical accuracy was evaluated by comparing MidP tumor locations to manually determined tumor displacements, while DIR precision was analyzed using the distance to discordance metric (DDM). Moreover, deformation vector fields (DVFs) from the DIR were used to automatically calculate MidP‐based treatment margins.

**Results:**

Eighteen targets were identified in seventeen patients. All HD‐MidP MR image sets were delineated, while 98% (53/54) of the SD‐MidP CT and 87% (47/54) of the SD‐MidP MR image sets were of adequate quality for delineation. The SD‐MidP MRI was positively scored in 13 out of 47 assessments for tumor distinctiveness and in 6 out of 47 assessments for OAR distinctiveness. In contrast, the HD‐MidP MRI showed a substantial improvement, with positive scores in 45 out of 54 assessments for tumor distinctiveness and 51 out of 54 assessments for OAR distinctiveness. Contour analyses revealed that the HD‐MidP MRI achieved the highest average DSC value (0.83) and, simultaneously, the lowest mean DTA value (0.96 mm). Compared to the manually determined tumor displacements, subvoxel differences in MidP tumor location were observed in 96% (52/54) of the registrations. The distribution of DDM values (median: 1.1 mm) for the HD‐MidP MRI was found to be significantly higher than the distributions for the SD‐MidP CT (median: 0.2 mm) and SD‐MidP MRI (median: 0.7 mm), indicating a lower, but still subvoxel, precision for the HD‐MidP MRI approach. The DVF variability was higher for the HD‐MidP MRI (median: 2.7 mm) than for the SD‐MidP MRI (median: 2.3 mm). However, when used to derive treatment margins, these margins were identical.

**Conclusions:**

The presented HD‐MidP MRI methodology scored highest on both tumor and OAR distinctiveness, with GTV contours demonstrating the best alignment. Combined with its high anatomical accuracy, these findings support its potential for lung radiotherapy planning.

## 1 INTRODUCTION

Breathing‐induced lung tumor motion introduces uncertainty in lung radiotherapy. To reduce the risk of target underdosage during free‐breathing treatments, large treatment margins are commonly applied.^1^, ^2^ The motion can be estimated with respiratory‐correlated four‐dimensional (4D) imaging,^3^, ^4^ where 4D computed tomography (CT) is the current standard of care to estimate patient‐specific tumor motion and to define or verify the internal target volume (ITV) and planning target volume (PTV).^5^, ^6^

However, in treatment planning, the use of an ITV approach can, depending on tumor motion, lead to substantial radiation exposure to healthy lung tissue. Mean lung dose has been recognized as the best predictor of grade ≥3 radiation pneumonitis.^7^ Among patients with non‐small‐cell lung cancer undergoing conventional chemoradiotherapy, pneumonitis rates can range from 15% to 40%. Those with interstitial lung disease are at even greater risk of developing pneumonitis and are particularly susceptible to even minor declines in pulmonary function.^8^ Recent studies have reported treatment‐related mortality rates between 7.9% and 15.6% following stereotactic radiotherapy in interstitial lung disease patients.^8^, ^9^ Moreover, the current standard of care for stage III lung cancer includes adjuvant immunotherapy, which has been associated with an additional risk of grade ≥2 pneumonitis — further emphasizing the importance of minimizing radiation exposure to healthy lung tissue.^10^ Oligometastatic lesions are increasingly treated with stereotactic radiotherapy during ongoing immunotherapy. While promising, the combined approach has been associated with a significantly higher incidence of grade 3 pneumonitis (10.7%) compared to stereotactic radiotherapy alone (0%).^11^

To reduce treatment volumes while maintaining target coverage, motion management such as respiratory gating or mid‐position (MidP)‐based planning can be used.^2^ The MidP image represents the time‐weighted average position of the tumor, created by deforming all respiratory phases to this average position using deformable image registration (DIR). This approach accounts for hysteresis in the 4D image set, the phenomenon that a tumor has a different motion path during inhalation and exhalation. Compared to individual phases, the MidP image better represents the average tumor position with reduced artifacts.^12^

Clinical validations have shown that using MidP CT images for PTV definition can reduce organ at risk (OAR) dose while maintaining target coverage.^13^, ^14^ In lung tissue, this effect is pronounced due to the wider beam penumbra. Nevertheless, the daily breathing variability can make the 4D‐CT an unreliable predictor for motion during treatment.^15^ While a verification 4D cone‐beam computed tomography (CBCT) can offer day‐of‐treatment motion information, it comes with exposure to additional ionizing radiation and it suffers from reduced image quality, especially for short scans. The latter can be prohibitive for MidP image generation.^16^

MRI offers superior soft‐tissue contrast without using ionizing radiation. While CT and CBCT effectively visualize pulmonary structures, MRI provides better anatomical visualization of the vasculature and soft tissues (e.g., mediastinum and heart).^17^ Moreover, integrating 4D‐MRI into the MRI‐simulation session helps to create motion‐managed treatment plans for abdominothoracic radiotherapy, either as a complement to 4D‐CT or within an MR‐only workflow. Nevertheless, CT remains the primary imaging modality for patient simulation, especially in the lung region, where MR imaging is challenging due to the low proton density. Although the development of ultrashort echo‐time MRI sequences has enabled the visualization of lung parenchyma,^18^, ^19^ these techniques are often limited by long acquisition and/or reconstruction times.

MR‐guided radiotherapy (MRgRT) enabled by integrated magnetic resonance (MR)‐linac systems supports online imaging and treatment adaptation.^20^, ^21^ Gated breath‐hold lung stereotactic radiotherapy has demonstrated excellent PTV coverage and OAR sparing on the 0.35 T MRIdian MR‐linac (Viewray Inc., Oakwood Village, Ohio, USA).^22^ However, inter‐breath‐hold beam‐off periods substantially increase treatment times,^23^ and controlling the target positioning during breath‐hold delivery may be challenging, especially for lung cancer patients.^24^ For MidP‐based treatments delivered in free‐breathing, 4D‐MRI techniques can be used to estimate anatomical motion during radiotherapy.^25^ By acquiring 4D‐MRI data on the day of treatment, the online MidP image and resulting PTV are obtained. Paulson et al.^26^ and van de Lindt et al.^13^ provided clinical evidence for the utility of 4D‐MRI for online adaptive abdominal MRgRT on the 1.5 T Unity MR‐linac (Elekta AB, Stockholm, Sweden). However, the acquisition of a 4D‐MRI for online adaptive MRgRT workflows has two main limitations: (1) the spatial resolution is typically lower compared to three‐dimensional (3D) scans because the temporal resolution has to be high enough to sample anatomical motion while maintaining an acceptable scan time, and (2) the flexibility to choose the desired MRI contrast may be limited.^27^

In this study, we comprehensively validate a hybrid high‐definition (HD)‐MidP MRI approach for lung radiotherapy planning. The first goal is to obtain MidP images with higher spatial resolution that are suitable for delineation, by combining 4D‐MRI motion information with a high‐resolution MRI. The second goal is to demonstrate the reliability of the DIR used to generate these MidP images, and its impact on deriving patient‐specific treatment margins.

## 2 METHODS

In this retrospective study, 25 patients with primary or metastatic lung tumors who underwent both CT‐ and MRI‐simulation between December 2020 and September 2022 were eligible and consecutively selected. Both CT and MRI data were acquired without contrast agent. Eight patients were excluded (details summarized in Table S1) due to poor MR image quality (*n* = 3), the use of an incorrect MRI protocol (*n* = 3), bulk motion exceeding 4 cm (*n* = 1) between MRI scans, or an incorrectly positioned field‐of‐view (FOV) (*n* = 1). The remaining 17 patients had complete imaging datasets and were treated with either conventional (*n* = 4) or stereotactic (*n* = 13) radiotherapy (details summarized in Table S2). Datasets were collected under the FAST‐ART protocol (IRB reference: 20‐519/C).

### 2.1 Image data

Four‐dimensional CT data were acquired on a Philips Brilliance Big Bore CT scanner (Best, The Netherlands) in free‐breathing to estimate respiratory‐induced motion for treatment planning. The FOV was 500–700×500–700×279–426 ${\mathrm{mm}}^{3}$ in the left‐right (LR), anterior‐posterior (AP), and cranial‐caudal (CC) directions, respectively. This dataset included 93–142 axial slices with an in‐plane resolution of typically 1.37×1.37 ${\mathrm{mm}}^{2}$ and a slice thickness of 3 mm. Phase binning based on the bellows surrogate signal sorted the data into a 4D‐CT with ten respiratory phases.

A simultaneous multi‐slice $T_{2}$‐weighted fast spin echo (FSE) 4D‐MRI research sequence was acquired on a Philips Ingenia 1.5 T scanner in free‐breathing to estimate respiratory‐induced motion during MRI‐simulation. The 4D‐MRI FOV was 457×208–338×350 ${\mathrm{mm}}^{3}$ (LR×AP×CC). This dataset included 52 coronal slices with an in‐plane resolution of 1.9×1.9 ${\mathrm{mm}}^{2}$ and a slice thickness varying between 4.0 and 6.5 mm. The image stack was acquired repeatedly for a total of 30 repetitions. Images were retrospectively sorted into ten amplitude bins that accounted for hysteresis, based on a self‐sorting signal derived from the liver‐lung interface through template matching.^28^ The 4D‐MR image sets were then corrected for spatial distortions caused by gradient nonlinearities using deformation vector fields (DVFs) derived from scanner‐specific spherical harmonics coefficients.^29^

In the same session, high‐resolution MRI data were acquired with an end‐exhale respiratory‐triggered axial multi‐slice $T_{2}$‐weighted FSE two‐dimensional MRI sequence.^30^ The imaging FOV was 500 × 500 × 140 ${\mathrm{mm}}^{3}$ (LR×AP×CC). This dataset included 40 axial slices with an in‐plane resolution of 0.5×0.5 ${\mathrm{mm}}^{2}$ and a slice thickness of 3.5 mm. When triggered by a one‐dimensional respiratory navigator,^31^ data were acquired for a period of 1029–1201 ms during the end‐exhale phase. The resulting 3D image was corrected offline for gradient nonlinearity distortions. Detailed scan explanation and parameters are summarized in Supplementary Section S1 and Table S3.

### 2.2 Mid‐position strategies

Two strategies to obtain a MidP image were evaluated in this work: (1) a standard‐definition (SD)‐MidP approach using only 4D image sets with the end‐exhale phase as reference image, and (2) an HD‐MidP approach combining motion information from 4D‐MR image sets with the high‐resolution MRI as the reference image to warp the latter to the MidP.

**FIGURE 1:**
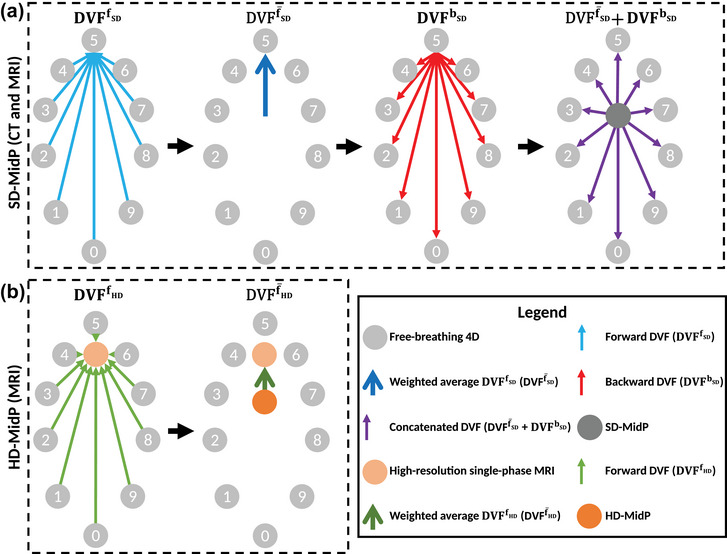
Illustration of the standard‐definition mid‐position (SD‐MidP) (a) and high‐definition (HD)‐MidP (b) approaches. The SD‐MidP approach uses forward deformation vector fields (${\mathrm{DVF}}^{f_{\mathrm{SD}}}$) and backward DVFs (${\mathrm{DVF}}^{b_{\mathrm{SD}}}$) to warp the ten respiratory phases to the MidP, whereas the HD‐MidP only requires forward DVFs (${\mathrm{DVF}}^{f_{\mathrm{HD}}}$) to warp the HD MRI to the MidP.

Figure 1a shows the three steps to obtain the SD‐MidP image.^25^ A vector pointing from phase A to B means that B was warped onto A, that is, the “pulling” method. First, DIR was performed from respiratory phases to the end‐exhale phase, generating nine forward DVFs (${\mathrm{DVF}}^{f_{\mathrm{SD}}}$). The time‐weighted average of the forward DVFs (${\mathrm{DVF}}^{{\bar{f}}_{\mathrm{SD}}}$) represents the DVF from the MidP to the reference image. The DVFs used to derive the MidP image from the phase‐binned 4D‐CT image sets had equal weights, as each respiratory phase contributed equally to the respiratory cycle. In contrast, the amplitude binning used for the 4D‐MR images resulted in an unequal distribution of images across amplitude bins. Therefore, each DVF of the 4D‐MR image set had a weight based on the relative contribution of each amplitude bin. Second, backward DVFs (${\mathrm{DVF}}^{b_{\mathrm{SD}}}$) were calculated and then weighted concatenated with the ${\mathrm{DVF}}^{{\bar{f}}_{\mathrm{SD}}}$, resulting in ten DVFs pointing from the MidP to the respiratory phases. This two‐step approach is required because of the implemented “pulling” method, which prevents areas of missing information in the warped image and allows for increasing the MidP image resolution.^32^ Third, the ten respiratory phases were warped to the MidP and the SD‐MidP image was obtained by calculating the weighted median voxel intensity.^28^ The SD‐MidP CT was calculated on the same voxel grid as the underlying respiratory phases, whereas the SD‐MidP MRI was calculated on a 1.9 × 1.9 × 1.9 ${\mathrm{mm}}^{3}$ grid. The through‐plane resolution (AP direction) of the latter could be increased relative to the 4D‐MRI scan because of complementary AP motion information in the sorted 4D‐MRI.

Figure 1b shows the method to obtain an HD‐MidP MRI. The ten respiratory phases of the 4D‐MRI were registered to the reference high‐resolution MRI. The time‐weighted average of these forward DVFs (${\mathrm{DVF}}^{{\bar{f}}_{\mathrm{HD}}}$) was used to warp the high‐resolution image to the MidP. The HD‐MidP MRI was calculated on a 0.5×0.5×1.2 ${\mathrm{mm}}^{3}$ grid, where the 1.2 mm matched the spatial slice selection resolution of our clinically used 3D workflow scan.

For the CT image sets, a clinically used optical flow DIR algorithm calculated the DVFs,^33^ using an intensity‐based formulation with an $L_{1}$ data fidelity term and $L_{2}$ spatial regularization (regularization parameter 0.3). Prior to registration, CT images were preprocessed using histogram equalization. The algorithm was executed twice to obtain both forward and backward DVFs. For the MR image sets, an intensity‐based and feature‐constrained DIR algorithm (Advanced Medical Imaging Registration Engine, research version 3.47, Elekta AB, Stockholm, Sweden) was used.^34^ The algorithm enforces inverse consistency by constraining concatenated forward and backward DVFs to be zero and was therefore executed once per image set. Further details are provided in Supplementary Section S2.

### 2.3 Clinical and technical validation

The tumor and overall OAR (e.g., esophagus, bronchi, aorta) distinctiveness in the MidP images were scored by three readers — consisting of two experienced radiation oncologists and one resident in radiation oncology — using a 4‐point Likert scale: 1 (“very poor”), 2 (“poor”), 3 (“good”), and 4 (“excellent”). A 4‐point scale was chosen to limit the number of options given the limited number of readers and to encourage a negative or positive assessment of image quality. Detailed scoring criteria are provided in Table S4. In addition, the gross tumor volume (GTV) was delineated on the MidP images in MIM 7.2.8 (MIM Software Inc., Cleveland, OH). A consensus GTV contour was derived from the union of pairwise intersections among the three contours per MidP image, serving as an estimate of the ground‐truth volume in the absence of clinical reference contours. The contours were analyzed by calculating the Dice similarity coefficient (DSC) and mean distance to agreement (DTA) between the individual contours and the consensus contour. Furthermore, the consensus GTV contour was used for the calculations of the distance discordance metric (DDM), the motion amplitude derived from the DVFs, and the 3D standard deviation (std) of the concatenated DVFs (Figure 1a) described below, by averaging over the voxels inside the GTV.

Tumor displacement was assessed manually by visually identifying the tumor in each respiratory phase using a DICOM viewer and recording its 3D image coordinates. The displacement relative to the reference image — the end‐exhale image set for the SD‐MidP approach and the high‐resolution MRI for the HD‐MidP approach — was then calculated from the coordinate differences. These displacements, discretized by the spatial resolution of the 4D image sets, provided a ground‐truth estimation of the motion amplitude, while the time‐weighted average displacement across all phases served as ground‐truth estimation of the MidP relative to the reference image. In addition, the tumor coordinates in the MidP image were determined to calculate the displacement between the MidP image and the reference image. This displacement was compared with the time‐weighted average displacement to calculate the systematic error component of DIR.

The random error component of the DIR used to obtain the MidP images was quantified using the DDM, based on the method introduced by van de Lindt et al.^25^ The DDM estimates voxel‐wise DIR uncertainty by measuring how much multiple registrations disagree about the location of the same anatomical point after warping. In 4D imaging, it quantifies how much the estimated position of a voxel varies when it is warped between images through different temporal registration paths using the same DIR, with lower DDM values indicating higher precision. First, DVFs were computed between the reference image and all intermediate images, excluding the end‐inhale image. Second, DVFs between each intermediate image and the end‐inhale image were determined. These DVFs were then concatenated, yielding a total of eight (SD‐MidP approach) or nine (HD‐MidP approach) DVFs that warp the end‐inhale image to the reference image. Third, these DVFs were concatenated with the DVF from the MidP image to the reference image (i.e., ${\mathrm{DVF}}^{\bar{f}}$), to project the DDM onto the MidP image.^28^ The DDM was determined by calculating the std of all DVFs per voxel location across the FOV.

The reliability of the DIR was also evaluated by extracting motion information from the DVFs used in the MidP approaches. The 3D motion amplitude of the tumor was derived from the DVFs and compared with the manually determined tumor displacements. In addition, the std of the DVFs that connected the 4D‐MRI phases to the MidP image was calculated voxel‐wise for each orthogonal direction (X, Y, and Z). These three stds per voxel were subsequently combined into a single 3D std. For the HD‐MidP approach, the backward DVF was determined to obtain the DVFs between the 4D‐MRI phases and the MidP image. In turn, the std of these DVFs was extracted. This std represents the amount of variability between the different DVFs and is less sensitive to outlier registrations compared to peak‐to‐peak estimates. Based on the std of the DVFs, patient‐specific anisotropic margins were determined using the nonlinear van Herk margin recipe, resulting in patient‐specific PTVs.^14^, ^35^ Further details can be found in Supplementary Section S3.

### 2.4 Statistics

A two‐sided Wilcoxon signed‐rank test with a 5% significance level was used to determine pairwise statistically significant differences between the generated MidP images for the image quality assessment, delineated GTVs, and the registration performance. A *p*‐value < 0.0167 was considered significant after Bonferroni correction (*m* = 3). The Spearman's rho test with a 5% significance level was used to evaluate correlations between the ordinal Likert scale scores and delineations, and between DDM values and 3D peak‐to‐peak motion. For the multiple pairwise comparisons of tumor volume agreement between readers, Bonferroni correction with a factor of 3 was applied.

## 3 RESULTS

The 4D‐MRI acquisition time was 4:01 min:s, while the mean (min–max) acquisition time for the high‐resolution MRI was 6:28 (4:51–8:15) min:s. The registration times for the MRI data were approximately 11–15 s (SD‐MidP MRI) and 30–35 s (HD‐MidP MRI). Calculating the MidP image took another 21–40 s.

### 3.1 Image quality

**FIGURE 2:**
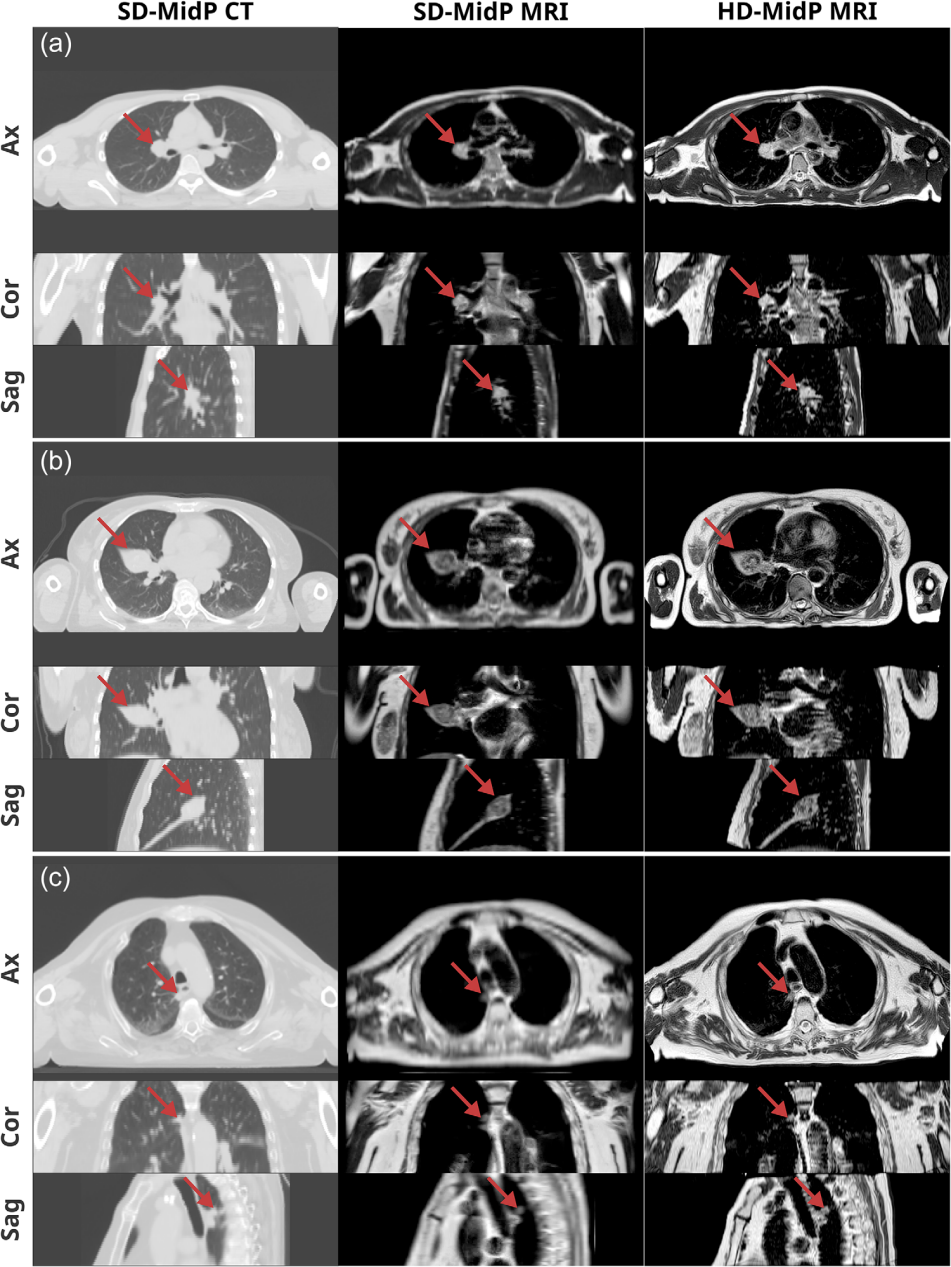
Examples of mid‐position (MidP) images of lung cancer patients for the standard‐definition (SD) and high‐definition (HD) approaches. The axial (Ax), coronal (Cor), and sagittal (Sag) planes intersecting the tumor location are shown. The arrows highlight the tumor location. The SD‐MidP CT is visualized using lung windowing (W:1700, L:−500), whereas MidP MR images have identical windowing (W:1860, L:1070). These examples were derived from 4D‐MRI scans with coronal slice thickness values of 4.0 mm (P2, a), 4.5 mm (P10, b), and 6.0 mm (P12, c).

Figure 2 shows examples of MidP images around the tumor location for three patients who were scanned with slice thicknesses of 4.0, 4.5, and 6.0 mm depending on their FOV in the AP direction. The MidP images were determined at the intersection of the two MRI FOVs, resulting in a FOV of 457 × 208–338 × 140 ${\mathrm{mm}}^{3}$ (LR×AP×CC). Figure S1 shows corresponding end‐exhale images for comparison.

**FIGURE 3:**
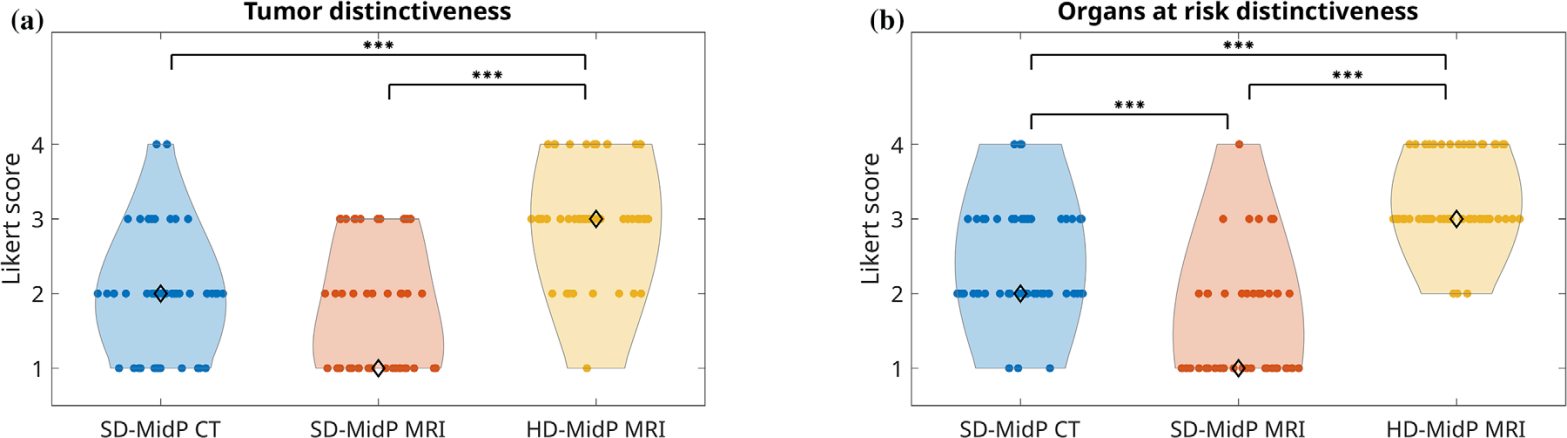
Image quality assessment results of the tumor and organs at risk distinctiveness for different mid‐position (MidP) images, with higher scores indicating better image quality. Three readers scored standard definition (SD)‐MidP CT and MR images, and high‐definition (HD)‐MidP MR images of eighteen targets. The diamond marker shows the modal value, that is, the most frequently occurring value. Significant differences are marked and indicate a *p*‐value below 0.00033 (***). Bonferroni correction was applied (*m* = 3).

Figure 3 shows the MidP image quality scores. SD‐MidP MR images of P5 and P6b were too blurry to differentiate the tumor and were therefore not scored and delineated. Additionally, both the SD‐MidP CT and SD‐MidP MRI of P17 were considered of insufficient quality by Reader #1. As a result, 53 out of 54 (98%) possible SD‐MidP CT scores and 47 out of 54 (87%) possible SD‐MidP MRI scores were obtained. The HD‐MidP MRI scored significantly (*p* < 0.00033) better than the SD‐MidP CT and SD‐MidP MR images for both the tumor and OAR distinctiveness. The modal values, that is, the most frequently occurring value, were 2 (SD‐MidP CT), 1 (SD‐MidP MRI), and 3 (HD‐MidP MRI). While for the tumor distinctiveness no significant difference was observed between SD‐MidP CT and SD‐MidP MRI (*p* = 0.14), the SD‐MidP MRI scored significantly (*p* < 0.00033) lower for the OAR distinctiveness. The SD‐MidP MRI showed “good” distinctiveness in 13 out of 47 assessed tumors, whereas the HD‐MidP MRI showed “good” or “excellent” distinctiveness in 45 out of 54 assessed tumors. The difference was even more pronounced for the OAR distinctiveness, with only 6 out of 47 SD‐MidP MR images being scored as “good” or “excellent” compared to 51 out of 54 for HD‐MidP MRI. The SD‐MidP MRI tumor distinctiveness (*p* < 0.0167) and SD‐MidP MRI OAR distinctiveness (*p* < 0.0033) were scored significantly higher by Reader #1 than by the other two readers, and the HD‐MidP MRI OAR distinctiveness was scored significantly higher (*p* < 0.0033) by Reader #1 compared with Reader #3. Kendall's coefficient of concordance indicated weak inter‐reader agreement (*W* = 0.22–0.34) across the MidP image quality scores.

### 3.2 Target delineations

Table S5 summarizes detailed tumor volumes. Spearman's rho test revealed strong agreement in the relative magnitude of tumor volumes across patients between Reader #1 and Reader #2 (ρ= 0.90, *p* < 0.00033), Reader #1 and Reader #3 (ρ= 0.94, *p* < 0.00033), and Reader #2 and Reader #3 (ρ= 0.91, *p* < 0.00033). Nevertheless, Reader #2 delineated smaller tumor volumes, whereas Reader #3 delineated larger volumes. The mean (std) consensus GTVs were 8.3 (8.0) cc, 7.5 (7.2) cc, and 6.5 (6.5) cc for SD‐MidP CT, SD‐MidP MR, and HD‐MidP MR images, respectively. No significant differences were observed between GTVs derived from SD‐MidP MR images and those from SD‐MidP CT (*p* = 0.13) or HD‐MidP MR (*p* = 0.26) images, while GTVs derived from SD‐MidP CT and HD‐MidP MR images were significantly different (*p* < 0.0167). On average, relative to the CT‐based GTVs, the MRI‐based GTVs were 15% (31% std) smaller on SD‐MidP MR and 17% (27% std) smaller on HD‐MidP MR images. Comparing individual contours of readers to the consensus contours, mean (std) DSC values of 0.83 (0.09), 0.81 (0.12), and 0.83 (0.13) were found for SD‐MidP CT, SD‐MidP MRI, and HD‐MidP MRI, respectively. The corresponding mean (std) DTA values were 0.9 (0.6), 1.0 (0.7), and 0.9 (0.8) mm. Among readers, Reader #2 exhibited the greatest disagreement with the consensus contour, reflected in significantly lower DSC (*p* < 0.00033) and higher DTA values (*p* < 0.00033). Further details are summarized in Tables S6 and S7. Likert scores were weakly to moderately positively correlated with DSC values for Reader #1 (ρ= 0.40, *p* < 0.01) and Reader #2 (ρ= 0.35, *p* < 0.05), both statistically significant. Additionally, for Reader #2, a significant weak negative correlation was observed between Likert scores and DTA values (ρ= −0.37, *p* < 0.01).

### 3.3 Registration performance

**FIGURE 4:**
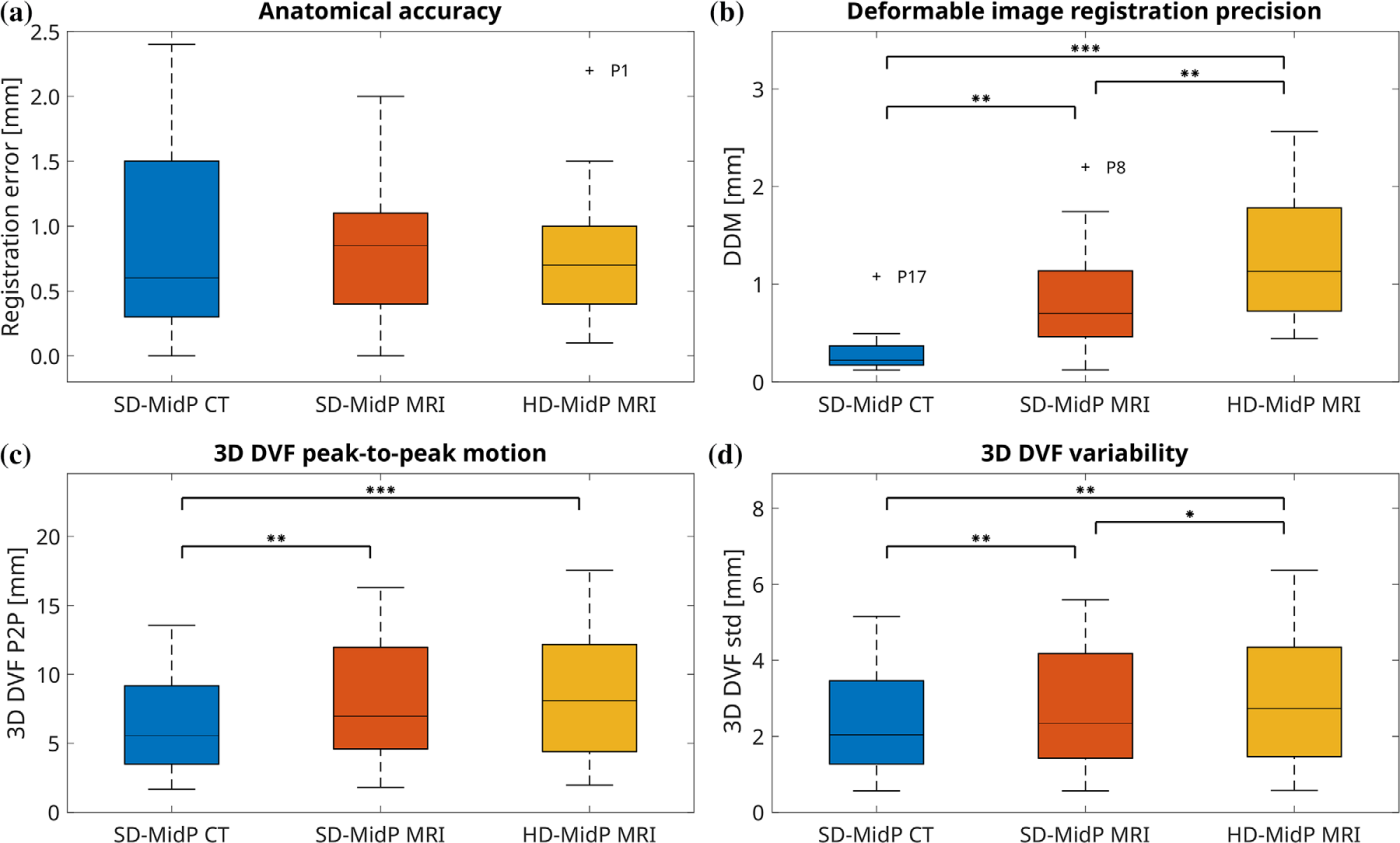
Quantitative results determined in the consensus gross tumor volume of the mid‐position (MidP) images: the anatomical accuracy of the MidP tumor location in the craniocaudal direction compared to the manually determined tumor displacement‐based position (a); lower distance discordance metric (DDM) values indicate higher deformable image registration precision (b); the 3D peak‐to‐peak (p2p) motion derived from the deformation vector fields (DVFs) (c); and the average 3D standard deviation (std) of the DVFs warping the respiratory phases to the MidP (d). Significant differences are marked and indicate *p*‐values below 0.0167 (*), 0.0033 (**), and 0.00033 (***), respectively. Bonferroni correction was applied (*m* = 3). Data outside the whiskers (1.5×interquartile range) were marked as outliers (+) and labeled with the patient number.

Figure 4a shows the anatomical accuracy defined as the error in CC direction between the manually determined tumor displacement‐based MidP locations and the MidP locations obtained with DIR. The median (25th–75th percentiles) differences were 0.6 (0.3–1.5) mm for SD‐MidP CT, 0.9 (0.4–1.1) mm for SD‐MidP MRI, and 0.7 (0.4–1.0) mm for HD‐MidP MRI. Subvoxel differences in MidP tumor location were observed in 52 out of 54 registrations (96%). Differences larger than the 4D‐MRI voxel size were found for the SD‐MidP MRI of P17 (2.0 mm) and the HD‐MidP MRI of P1 (2.2 mm).

Figure 4b shows the distributions of average DDM values within the consensus GTV. The median (25th–75th percentiles) DDM values were 0.2 (0.2–0.4) mm for SD‐MidP CT, 0.7 (0.5–1.1) mm for SD‐MidP MRI, and 1.1 (0.7–1.8) mm for HD‐MidP MRI. The distributions of DDM values for the MidP MRI registrations were significantly higher than the SD‐MidP CT registrations (*p* < 0.0033). The HD‐MidP MRI registrations had a significantly higher DDM than the SD‐MidP MRI registrations (*p* < 0.0033). One SD‐MidP and four HD‐MidP MRI registrations had a DDM value larger than the 4D‐MRI in‐plane resolution: SD‐MidP of P8, and the HD‐MidP of P1, P8, P12, and P17, with P8 and P17 having the largest motion amplitude among the patients.

Figure 4c shows the distribution of 3D peak‐to‐peak amplitudes and Figure 4d shows the distribution of variability in the 3D DVFs that linked the MidP image to the 4D respiratory phases. Median (25th–75th percentiles) amplitudes were 5.6 (3.5–9.2), 7.0 (4.6–12.0), and 8.1 (4.4–12.2) mm for SD‐MidP CT, SD‐MidP MRI, and HD‐MidP MRI, respectively. The amplitudes derived from the DVFs of the CT image sets were significantly smaller compared to the SD‐MidP MRI (*p* < 0.0033) and HD‐MidP MRI (*p* < 0.00033). Compared to manually determined tumor displacement‐based translations, mean absolute differences in 3D peak‐to‐peak tumor motion were 1.5 mm (SD‐MidP CT), 1.3 mm (SD‐MidP MRI), and 1.2 mm (HD‐MidP MRI). Significant positive Spearman's correlations were found between the DDM values and the 3D peak‐to‐peak motion amplitudes for SD‐MidP CT (ρ= 0.83, *p* < 0.0001), SD‐MidP MRI (ρ= 0.73, *p* < 0.01), and HD‐MidP MRI (ρ= 0.74, *p* < 0.0001). Although the distributions of DVF variability were similar for the SD‐MidP MRI (median: 2.3 mm, 25th–75th percentiles: 1.4–4.2 mm) and HD‐MidP MRI (median: 2.7 mm, 25th–75th percentiles: 1.5–4.3 mm) registrations, the difference was significant (*p* < 0.0167). Margins derived using this DVF motion information, which is further described in Supplementary Section S3, were identical for the two MRI approaches. These margins resulted in mean (std) PTVs of 18.4 (14.4) cc, 17.2 (13.2) cc, and 15.6 (11.9) cc for respectively the SD‐MidP CT, SD‐MidP MRI, and HD‐MidP MRI (Table S8).

## 4 DISCUSSION

In this study, we validated a novel HD‐MidP MRI methodology for lung radiotherapy planning, which improves the MidP spatial resolution and enables the generation of MidP images with a different image contrast and acquisition orientation compared to the 4D‐MRI. Our study showed that HD‐MidP MRI significantly outperformed SD‐MidP CT and SD‐MidP MRI on image quality and had the best agreement across GTV delineations; however, the GTVs derived from HD‐MidP MR images were statistically significantly smaller than those derived from SD‐MidP CT images. DIR validation revealed minor differences between the SD‐MidP and HD‐MidP MRI approaches, which did not impact the patient‐specific margins. By combining precise motion estimation and high‐quality MR imaging with patient‐specific margins, our approach has the potential to enhance treatment accuracy while minimizing exposure to normal tissues. It opens opportunities for advancements in MR‐guided lung radiotherapy workflows and is expected to be translatable to upper‐abdominal radiotherapy as well.

High‐resolution MRI data were acquired with a respiratory‐triggered 2D‐MRI sequence. After triggering the imaging sequence, data were acquired for a period of 1029–1201 ms, independent of the breathing frequency. For patients with long breathing cycles, this approach increases total scan time and motion artifact risk due to the number of respiratory triggers required. This inefficiency contributed to scan times of up to 8:15 min:s. Scan efficiency could be improved by acquiring data during the entire end‐exhale phase and/or using (audio)‐visual biofeedback.^36^ This could also reduce the risk of stitching artifacts in slice‐based respiratory‐triggered images, which can result from incorrect triggering or prolonged acquisition times and were responsible for exclusions in this study.

For the 4D‐MRI data acquisition, coronal slice thicknesses were adjusted based on the anatomy (4.0–6.5 mm) to ensure consistent dataset dimensions across patients. Despite the coarse slice thickness, the DIR precision quantification revealed median DDM values of 0.7 mm (SD‐MidP MRI) and 1.1 mm (HD‐MidP MRI), consistent with previous findings for MidP liver radiotherapy.^25^ The higher, but nonetheless small, DDM values for the HD‐MidP approach are likely a result of the difference in voxel size and acquisition orientation of the two MRI acquisitions, but no differences in margins were observed. Coarse slice thickness in 4D‐MRI may obscure anatomical structures that are clearly visible in high‐resolution images. Therefore, it might be advisable to change the 4D‐MRI acquisition to the axial orientation combined with a constant slice thickness and variable number of slices. However, this approach complicates the self‐sorting signal extraction due to the main component of the respiratory motion being in the through‐plane direction and increases the total scan time. A method to extract a self‐sorting signal from axial liver images exists,^37^ but for lung radiotherapy this is more difficult due to the lack of moving reference structures. Alternatively, the (axial) 4D‐MRI acquisition can be combined with a one‐dimensional respiratory navigator acquisition to obtain a surrogate signal.^38^ This can be combined with visual biofeedback, which helps to maintain stable and regular breathing, thereby minimizing intra‐fraction changes in the breathing pattern.^39^ As a result, the number of stack acquisition repetitions could possibly be reduced while maintaining good‐quality 4D‐MR image sets.^25^ Another solution could be to use a 3D‐MRI sequence for 4D‐MRI data acquisition, but such sequences typically result in ($T_{2}$/)$T_{1}$‐weighted images; however, for our validated method, the 4D‐MR image sets are used to estimate the DVFs and corresponding MidP image, and therefore the contrast is not a limitation. This has previously been demonstrated by Freedman et al.,^40^ where $T_{2}$‐weighted 4D‐MR image sets were obtained using DIR. Furthermore, using a 3D‐MRI‐based 4D‐MRI sequence could improve the visibility of small tumors or tumors near the heart, potentially preventing exclusions. In this study, two patients with small tumors near the left ventricle were excluded because the tumor was not consistently visualized across all 4D‐MRI phases. This was likely due to the coarse spatial resolution and the acquisition being respiratory‐correlated without cardiac motion compensation. A five‐dimensional MRI sequence could mitigate both respiratory and cardiac motion, but would likely require additional acquisition or reconstruction time.

The image quality was assessed using a 4‐point Likert scale to verify the clinical usability of the MidP images. Figure S1 presents representative end‐exhale images, demonstrating the high level of anatomical detail achieved with the respiratory‐triggered MRI acquisition. The SD‐MidP MRI scored the lowest on both tumor and OAR distinctiveness due to MidP image blurring in the preferred axial plane caused by the coronal 4D‐MRI acquisition. Compared to the SD‐MidP MRI approach based on coarser voxels, the HD‐MidP MRI approach significantly improved image quality by leveraging high‐resolution MRI data and was scored better than the SD‐MidP CT images. This finding for these (ultra‐)central tumors is of high clinical interest, as severe toxicity rates of up to 35% have been reported in patients with central lung tumors.^41^ Notably, doses delivered to the bronchi have been identified as the primary risk factor for such toxicity.^42^, ^43^ Therefore, margin reduction may hold critical clinical relevance for this patient group. Recent analyses of MRgRT in patients with central tumors have shown promisingly low toxicity rates, highlighting the potential of this approach.^44^ Interestingly, our study found that MRI‐based GTVs were on average 17% smaller compared to CT‐based GTVs. Similarly, Karki et al. (7 readers, 10 patients) reported smaller lung tumor volumes on MRI, with CT‐ and MRI‐based volumes being 1.62 and 1.38 times larger than PET‐CT‐based volumes, respectively, which indicates a 15% difference between CT and MRI.^45^ Likewise, Hall et al. (12 readers, 3 patients) reported 20% (57.48 vs. 45.76 cc) smaller MRI‐based volumes for pancreatic cancer.^46^ Although CT is commonly regarded as the reference standard, it does not necessarily represent the ground‐truth. While average differences of 15/17% were observed between CT‐ and MRI‐based GTVs in this study, no pathological or surgical ground‐truth was available to determine which modality more accurately represented the true tumor volume. Further investigation, including comparison with pathologic measurements, is required to accurately define the GTV that avoids systematic over‐ or underestimation of the tumor region.

Validation of anatomical accuracy through manually determined tumor displacements revealed that systematic errors in the DIR process were smaller than 2 mm in 96% of cases, with median errors of 0.6–0.9 mm, which is within the commonly accepted 2 mm accuracy threshold for clinical applications.^47^ This systematic error, together with the random DIR error, can be accounted for in the van Herk margin recipe used in this work, resulting in a margin expansion of 1–3 mm (see Supplementary Section S3). For (ultra‐)central tumors, this margin expansion can affect the treatment planning process when critical OARs are nearby, potentially leading to decreased dose levels in the GTV and/or increased radiation exposure to healthy tissue, which should be minimized to limit the risk of pneumonitis. Despite the small systematic errors, the manually determined tumor displacements had their limitations. The focus was on the tumor motion in CC direction, with spatial resolutions of 3.0 mm (4D‐CT) and 1.9 mm (4D‐MRI), limiting the accuracy in defining the ground‐truth MidP location. Additionally, determining motion in the AP direction was challenging for MRI datasets with larger slice thicknesses, especially for smaller tumors. Nevertheless, the MidP‐PTV margins using manually determined tumor displacements were identical to those based on DVFs.

The reliability assessment of the DVFs revealed only minor differences in motion information for the MidP MRI methods, indicating that changing the reference image had a minimal effect on the DIR performance. A pre‐processing step performing rigid registration could be implemented in future studies to account for bulk motion and further reduce DIR errors. As this analysis was performed using a consistent 4D‐MRI and high‐resolution MRI protocol and excluded datasets with acquisition inconsistencies, the current implementation is expected to perform best within a standardized clinical workflow with fixed acquisition settings across patients. For clinical introduction, it is advisable to output one or multiple quantitative metrics along with the MidP image to indicate the DIR performance,^47^ similarly to MIM,^48^ which is clinically used by Paulson et al.^26^ to derive MidP MR images. The analysis of the DVFs also showed that the anatomical motion encoded in the CT and MR image sets was significantly different, highlighting the importance of estimating the motion at the day of treatment and adjusting the treatment plan accordingly.

In the future, the HD‐MidP MRI approach could be combined with ultrashort echo‐time MR images for improved visualization of the lung parenchyma and/or potentially support MR‐only workflows, the latter reducing patient logistics and minimizing uncertainties introduced by CT‐to‐MRI registration.^49^ Moreover, the need for the approach could be determined based on tumor motion amplitude.^26^ When tumor motion is small (e.g., less than 8 mm), an average intensity projection image could be used. This simplifies the workflow and reduces uncertainties, as DIR is not required (see Supplementary Section S4 and Figure S3 for explanation and examples).

The validated method has potential to support MR‐guided radiotherapy workflows on an MR‐linac, but its immediate translatability is limited by the fact that the MRI data were acquired on an MRI‐simulator. In addition, the specific 4D‐MRI research sequence used in this study is currently not part of the clinical workflow at the Unity MR‐linac. To obtain the motion information of the day, the 4D‐MRI scan must be acquired and, to minimize on‐table time, it might be undesirable to also acquire the respiratory‐triggered scan during an online workflow, as the protocol used in this study required on average 10:29 min:s (4:01 + 6:28) of acquisition time, while the MidP reconstruction (performed offline) required approximately one minute. This limitation can be overcome by using the triggered MRI from the MRI‐simulation session and acquiring the 4D‐MRI only as a daily scan, as the sorting of a slice‐based 4D‐MRI can be performed in seconds. However, anatomical changes between MRI‐simulation and the first treatment session could affect registration accuracy, though this should be captured by a metric like DDM. Future work should focus on validating the feasibility of combining high‐resolution single‐phase MR images acquired during MRI‐simulation with 4D‐MRI datasets acquired on a different day on an MR‐linac, and to perform a dosimetric comparison between treatment plans based on the different MidP images. Moreover, practical implementation on an MR‐linac will require optimization to ensure that 4D‐MRI acquisition and processing, DIR, and final HD‐MidP reconstruction fit within the time frame of an adaptive MR‐linac workflow for plan adaptation. In addition to using the generated HD‐MidP MRI for plan adaptation, it also has potential for use as a reference image for real‐time motion monitoring during gated deliveries.^50^ Furthermore, the use of the approach for upper‐abdominal radiotherapy should be investigated, as well as the DIR performance for 4D image sets and high‐resolution images with different MRI contrast.

## 5 CONCLUSIONS

The presented methodology obtains an HD‐MidP MRI with high geometric fidelity suitable for lung radiotherapy planning. The required DIR was found to be accurate and provided essential information for automatically deriving margins for a MidP‐based treatment plan. The observed differences in breathing‐induced tumor motion between the CT and MRI datasets, which were acquired at different times, highlight the importance of estimating the motion prior to treatment. Overall, this work indicates the suitability and relevance of the presented methodology for MR‐guided lung radiotherapy workflows.

## CONFLICT OF INTEREST STATEMENT

The authors declare no conflicts of interest.

## Supporting information

Supporting Information

## References

[1] Keall PJ , Mageras GS , Balter JM , et al. The management of respiratory motion in radiation oncology report of AAPM Task Group 76. Med Phys. 2006;33(10):3874‐3900. doi: 10.1118/1.2349696 17089851

[2] Wolthaus JW , Sonke JJ , van Herk M , et al. Comparison of different strategies to use four‐dimensional computed tomography in treatment planning for lung cancer patients. Int J Radiat Oncol Biol Phys. 2008;70(4):1229‐1238. doi: 10.1016/j.ijrobp.2007.11.042 18313530

[3] Ford EC , Mageras GS , Yorke E , Ling CC . Respiration‐correlated spiral CT: a method of measuring respiratory‐induced anatomic motion for radiation treatment planning. Med Phys. 2003;30(1):88‐97. doi: 10.1118/1.1531177 12557983

[4] Sonke JJ , Zijp L , Remeijer P , van Herk M . Respiratory correlated cone beam CT. Med Phys. 2005;32(4):1176‐1186. doi: 10.1118/1.1869074 15895601

[5] Keall P . 4‐dimensional computed tomography imaging and treatment planning. Semin Radiat Oncol. 2004;14(1):81‐90. doi: 10.1053/j.semradonc.2003.10.006 14752736

[6] Underberg RW , Lagerwaard FJ , Cuijpers JP , Slotman BJ , De Koste JRVS , Senan S . Four‐dimensional CT scans for treatment planning in stereotactic radiotherapy for stage I lung cancer. Int J Radiat Oncol Biol Phys. 2004;60(4):1283‐1290. doi: 10.1016/j.ijrobp.2004.07.665 15519801

[7] Harder EM , Park HS , Chen ZJ , Decker RH . Pulmonary dose‐volume predictors of radiation pneumonitis following stereotactic body radiation therapy. Pract Radiat Oncol. 2016;6(6):e353‐e359. doi: 10.1016/j.prro.2016.01.015 27156424

[8] Chen H , Senan S , Nossent EJ , et al. Treatment‐related toxicity in patients with early‐stage non‐small cell lung cancer and coexisting interstitial lung disease: a systematic review. Int J Radiat Oncol Biol Phys. 2017;98(3):622‐631. doi: 10.1016/j.ijrobp.2017.03.010 28581404

[9] Li GJ , Soon MS , Chen H , et al. Treatment toxicity and outcomes following definitive radiotherapy for patients with early‐stage non‐small cell lung cancers and pre‐existing interstitial lung disease–a systematic review and dosimetric analysis. Int J Radiat Oncol Biol Phys. 2025;123(4):1050‐1060. doi: 10.1016/j.ijrobp.2025.06.3854 40581332

[10] Stoffers RH , Niezink AG , Chouvalova O , et al. Rising incidence of radiation pneumonitis after adjuvant durvalumab in NSCLC patients treated with concurrent chemoradiotherapy. Acta Oncol. 2025;64:42384. doi: 10.2340/1651-226X.2025.42384 39945611 PMC11848943

[11] Tian S , Switchenko JM , Buchwald ZS , et al. Lung stereotactic body radiation therapy and concurrent immunotherapy: a multicenter safety and toxicity analysis. Int J Radiat Oncol Biol Phys. 2020;108(1):304‐313. doi: 10.1016/j.ijrobp.2019.12.030 31982496 PMC7747230

[12] Wolthaus JWH , Sonke JJ , van Herk M , Damen EMF . Reconstruction of a time‐averaged midposition CT scan for radiotherapy planning of lung cancer patients using deformable registration. Med Phys. 2008;35(9):3998‐4011. doi: 10.1118/1.2966347 18841851

[13] van de Lindt TN , Nowee ME , Janssen T , et al. Technical feasibility and clinical evaluation of 4D‐MRI guided liver SBRT on the MR‐linac. Radiother Oncol. 2022;167:285‐291. doi: 10.1016/j.radonc.2022.01.009 35033603

[14] Ligtenberg H , Hackett SL , Merckel LG , et al. Towards mid‐position based stereotactic body radiation therapy using online magnetic resonance imaging guidance for central lung tumours. Phys Imaging Radiat Oncol. 2022;23:24‐31. doi: 10.1016/j.phro.2022.05.002 35923896 PMC9341269

[15] Dhont J , Vandemeulebroucke J , Burghelea M , et al. The long‐and short‐term variability of breathing induced tumor motion in lung and liver over the course of a radiotherapy treatment. Radiother Oncol. 2018;126(2):339‐346. doi: 10.1016/j.radonc.2017.09.001 28992962

[16] Rit S , Nijkamp J , van Herk M , Sonke JJ . Comparative study of respiratory motion correction techniques in cone‐beam computed tomography. Radiother Oncol. 2011;100(3):356‐359. doi: 10.1016/j.radonc.2011.08.018 21924782

[17] Noel CE , Parikh PJ , Spencer CR , et al. Comparison of onboard low‐field magnetic resonance imaging versus onboard computed tomography for anatomy visualization in radiotherapy. Acta Oncol. 2015;54(9):1474‐1482. doi: 10.3109/0284186X.2015.1062541 26206517

[18] Dournes G , Menut F , Macey J , et al. Lung morphology assessment of cystic fibrosis using MRI with ultra‐short echo time at submillimeter spatial resolution. Eur Radiol. 2016;26:3811‐3820. doi: 10.1007/s00330-016-4218-5 26843010

[19] Wu C , Krishnamoorthy G , Yu V , Subashi E , Rimner A , Otazo R . 4D lung MRI with high‐isotropic‐resolution using half‐spoke (UTE) and full‐spoke 3D radial acquisition and temporal compressed sensing reconstruction. Phys Med Biol. 2023;68(3):035017. doi: 10.1088/1361-6560/acace6 PMC10257135 36535035

[20] Olsen J , Green O , Kashani R . World's first applicaton of MR‐guidance for radiotherapy. Mo Med. 2015;112(5):358‐360. 26606816 PMC6167237

[21] Raaymakers BW , Jürgenliemk‐Schulz IM , Bol GH , et al. First patients treated with a 1.5 T MRI‐Linac: clinical proof of concept of a high‐precision, high‐field MRI guided radiotherapy treatment. Phys Med Biol. 2017;62(23):L41‐L50. doi: 10.1088/1361-6560/aa9517 29135471

[22] Finazzi T , Haasbeek CJA , Spoelstra FOB , et al. Clinical outcomes of stereotactic MR‐guided adaptive radiation therapy for high‐risk lung tumors. Int J Radiat Oncol Biol Phys. 2020;107(2):270‐278. doi: 10.1016/j.ijrobp.2020.02.025 32105742

[23] Ehrbar S , Käser SB , Chamberlain M , et al. MR‐guided beam gating: residual motion, gating efficiency and dose reconstruction for stereotactic treatments of the liver and lung. Radiother Oncol. 2022;174:101‐108. doi: 10.1016/j.radonc.2022.07.007 35839937

[24] Tetar S , Bruynzeel A , Bakker R , et al. Patient‐reported outcome measurements on the tolerance of magnetic resonance imaging‐guided radiation therapy. Cureus. 2018;10(2):e2236. doi: 10.7759/cureus.2236 29719739 PMC5922504

[25] van de Lindt T , Fast M , Van Kranen S , et al. MRI‐guided mid‐position liver radiotherapy: validation of image processing and registration steps. Radiother Oncol. 2019;138:132‐140. doi: 10.1016/j.radonc.2019.06.007 31252295

[26] Paulson ES , Ahunbay E , Chen X , et al. 4D‐MRI driven MR‐guided online adaptive radiotherapy for abdominal stereotactic body radiation therapy on a high field MR‐Linac: implementation and initial clinical experience. Clin Transl Radiat Oncol. 2020;23:72‐79. doi: 10.1016/j.ctro.2020.05.002 32490218 PMC7256110

[27] Stemkens B , Paulson ES , Tijssen RHN . Nuts and bolts of 4D‐MRI for radiotherapy. Phys Med Biol. 2018;63(21):21TR01. doi: 10.1088/1361-6560/aae56d 30272573

[28] Keijnemans K , Borman PTS , van Lier ALHMW , Verhoeff JJC , Raaymakers BW , Fast MF . Simultaneous multi‐slice accelerated 4D‐MRI for radiotherapy guidance. Phys Med Biol. 2021;66(9):095014. doi: 10.1088/1361-6560/abf591 33827065

[29] Keesman R , van de Lindt TN , Juan‐Cruz C , et al. Correcting geometric image distortions in slice‐based 4D‐MRI on the MR‐linac. Med Phys. 2019;46(7):3044‐3054. doi: 10.1002/mp.13602 31111494

[30] Pipe JG . Motion correction with PROPELLER MRI: application to head motion and free‐breathing cardiac imaging. Magn Reson Med. 1999;42(5):963‐969. doi: 10.1002/(SICI)1522-2594(199911)42:5003C;963::AID-MRM17003E;3.0.CO;2-L 10542356

[31] Ehman RL , Felmlee JP . Adaptive technique for high‐definition MR imaging of moving structures. Radiology. 1989;173(1):255‐263. doi: 10.1148/radiology.173.1.2781017 2781017

[32] Murr M , Brock KK , Fusella M , et al. Applicability and usage of dose mapping/accumulation in radiotherapy. Radiother Oncol. 2023;182:109527. doi: 10.1016/j.radonc.2023.109527 36773825 PMC11877414

[33] Zachiu C , Papadakis N , Ries M , Moonen C , De Senneville BD . An improved optical flow tracking technique for real‐time MR‐guided beam therapies in moving organs. Phys Med Biol. 2015;60(23):9003. doi: 10.1088/0031-9155/60/23/9003 26540256

[34] Juan‐Cruz C , Fast MF , Sonke JJ . A multivariable study of deformable image registration evaluation metrics in 4DCT of thoracic cancer patients. Phys Med Biol. 2021;66(3):035019. doi: 10.1088/1361-6560/abcd18 33227717

[35] van Herk M , Remeijer P , Rasch C , Lebesque JV . The probability of correct target dosage: dose‐population histograms for deriving treatment margins in radiotherapy. Int J Radiat Oncol Biol Phys. 2000;47(4):1121‐1135. doi: 10.1016/S0360-3016(00)00518-6 10863086

[36] Lee D , Greer PB , Arm J , Keall P , Kim T . Audiovisual biofeedback improves image quality and reduces scan time for respiratory‐gated 3D MRI. J Phys Conf Ser. 2014;489(1):012033. doi: 10.1088/1742-6596/489/1/012033

[37] van de Lindt TN , Fast MF , van der Heide UA , Sonke JJ . Retrospective self‐sorted 4D‐MRI for the liver. Radiother Oncol. 2018;127(3):474‐480. doi: 10.1016/j.radonc.2018.05.006 29804801

[38] Li G , Wei J , Olek D , et al. Direct comparison of respiration‐correlated four‐dimensional magnetic resonance imaging reconstructed using concurrent internal navigator and external bellows. Int J Radiat Oncol Biol Phys. 2017;97(3):596‐605. doi: 10.1016/j.ijrobp.2016.11.004 28011048 PMC5288126

[39] Keijnemans K , Borman PT , Raaymakers BW , Fast MF . Effectiveness of visual biofeedback–guided respiratory‐correlated 4D‐MRI for radiotherapy guidance on the MR‐linac. Magn Reson Med. 2023;91(1):297‐311. doi: 10.1002/mrm.29857 37799101

[40] Freedman JN , Collins DJ , Bainbridge H , et al. T2‐weighted 4D magnetic resonance imaging for application in magnetic resonance–guided radiotherapy treatment planning. Invest Radiol. 2017;52(10):563‐573. doi: 10.1097/RLI.0000000000000381 28459800 PMC5581953

[41] Tekatli H , Giraud N , van Eekelen R , Lagerwaard FJ , Senan S . Ten years outcomes after SABR in central and ultracentral primary lung tumors. Radiother Oncol. 2023;188:109848. doi: 10.1016/j.radonc.2023.109848 37562553

[42] Lindberg K , Grozman V , Karlsson K , et al. The HILUS‐trial—A prospective nordic multicenter phase 2 study of ultracentral lung tumors treated with stereotactic body radiotherapy. J Thorac Oncol. 2021;16(7):1200‐1210. doi: 10.1016/j.jtho.2021.03.019 33823286

[43] Lindberg S , Grozman V , Karlsson K , et al. Expanded HILUS trial: a pooled analysis of risk factors for toxicity from stereotactic body radiation therapy of central and ultracentral lung tumors. Int J Radiat Oncol Biol Phys. 2023;117(5):1222‐1231. doi: 10.1016/j.ijrobp.2023.06.246 37423292

[44] Tekatli H , Giraud N , Palacios MA , et al. 1787: MR‐guided stereotactic ablative radiotherapy for central lung tumors: long‐term clinical outcomes. Radiother Oncol . 2024;194, Supplement 1:S1695–S1697. doi: 10.1016/S0167-8140(24)02121-2 26472316

[45] Karki K , Saraiya S , Hugo GD , et al. Variabilities of magnetic resonance imaging–, computed tomography–, and positron emission tomography–computed tomography–based tumor and lymph node delineations for lung cancer radiation therapy planning. Int J Radiat Oncol Biol Phys. 2017;99(1):80‐89. doi: 10.1016/j.ijrobp.2017.05.002 28816167 PMC5607632

[46] Hall WA , Heerkens HD , Paulson ES , et al. Pancreatic gross tumor volume contouring on computed tomography (CT) compared with magnetic resonance imaging (MRI): results of an international contouring conference. Pract Radiat Oncol. 2018;8(2):107‐115. doi: 10.1016/j.prro.2017.11.005 29426692

[47] Brock KK , Mutic S , McNutt TR , Li H , Kessler ML . Use of image registration and fusion algorithms and techniques in radiotherapy: report of the AAPM radiation therapy committee task group No. 132. Med Phys. 2017;44(7):e43‐e76. doi: 10.1002/mp.12256 28376237

[48] Rong Y , Rosu‐Bubulac M , Benedict SH , et al. Rigid and deformable image registration for radiation therapy: a self‐study evaluation guide for NRG oncology clinical trial participation. Pract Radiat Oncol. 2021;11(4):282‐298. doi: 10.1016/j.prro.2021.02.007 33662576 PMC8406084

[49] Owrangi AM , Greer PB , Glide‐Hurst CK . MRI‐only treatment planning: benefits and challenges. Phys Med Biol. 2018;63(5):05TR01. doi: 10.1088/1361-6560/aaaca4 PMC5886006 29393071

[50] Jassar H , Tai A , Chen X , et al. Real‐time motion monitoring using orthogonal cine MRI during MR‐guided adaptive radiation therapy for abdominal tumors on 1.5 T MR‐Linac. Med Phys. 2023;50(5):3103‐3116. doi: 10.1002/mp.16342 36893292

