# Supplementary material for: Clinical validation of a high‐definition mid‐position magnetic resonance imaging approach for lung radiotherapy planning
Source: Med Phys. 2026 May 5;53:e70457. doi: 10.1002/mp.70457 (PMC13143559; doi:10.1002/mp.70457)
Supplement: Supplementary file 1 — Supporting Information [file MP-53-0-s001.pdf]

Excluded patient datasets

Table S1: Summary of patient exclusion criteria.

**Exclusion category**	**Reason for exclusion**
Poor image quality (n=3)	The tumor was not visible in the four-dimensional magnetic resonance imaging (4D-MRI) data (n=2).<br>A stitching artifact was present in the reconstructed respiratory-triggered high-resolution MRI (n=1).
Incorrect MRI protocol (n=3)	A fat-suppressed respiratory-triggered high-resolution MRI scan with a different matrix dimension was acquired (n=2).<br>A respiratory-triggered high-resolution MRI scan with a different matrix dimension was acquired, which also presented with a stitching artifact (n=1).
Bulk motion (n=1)	Patient bulk motion larger than 4 cm was observed between the two MRI acquisitions (n=1).
Incorrect positioned field-of-view (n=1)	The tumor was only partially included in the acquired respiratory-triggered high-resolution MRI data (n=1).

Patient characteristics

Table S2: Summary of the patient characteristics and treatment fractionation delivered using either a cone-beam computed tomography guided linear accelerator (CBCT-linac) or a magnetic resonance imaging guided linear accelerator (MR-linac). Note that the 17 patients had 18 targets in total.

Sex	
Male	12
Female	5
Weight [kg]	
min–median–max	50–86–118
Age [years]	
min–median–max	51–62–79
Target type	
Primary lung tumor	10
Metastatic lung tumor	8
Primary tumor location	
Lung	10
Kidney	3
Colon	2
Thyroid	1
Esophagus	1
Skin	1
Target location	
Ultra-central[a]	8
Central[b]	9
Peripheral	1
Treatment	
12×3.5 Gy MR-linac	1
12×5 Gy MR-linac	7
8×7.5 Gy MR-linac	5
25×2.6 Gy CBCT-linac	2
18×3 Gy CBCT-linac	1
13×3 Gy CBCT-linac	1
12×5 Gy CBCT-linac	1

a) Located within 1 cm of the proximal bronchial tree (Lindberg et al., 2021, J. Thorac. Oncol.). b) Located within 2 cm of any critical mediastinal structure according to the International Association for the Study of Lung Cancer (Chang et al., 2015, J Thorac Oncol.).

S1. Data acquisition details

The high-resolution MRI data were acquired with a Periodically Rotated Overlapping ParallEL Lines with Enhanced Reconstruction (PROPELLER) imaging sequence. When triggered by the one-dimensional respiratory navigator (1D-RNAV), the PROPELLER sequence acquired a blade (i.e., several parallel phase-encoded radial readouts) at 6–7 slice locations during the end-exhale phase, which was rotated for subsequent readouts. The acquisition time of a blade was 172 ms. The 1D-RNAV was acquired on the liver-lung interface with a 100 mm length in cranial-caudal and a diameter of 30 mm. The 1D-RNAV was acquired every 200 ms, and it triggered the imaging sequence to acquire image data based on the velocity of the liver-lung interface during exhalation.

Table S3: Summary of the scan parameters for the four-dimensional computed tomography (4D-CT), 4D- magnetic resonance imaging (MRI), and respiratory-triggered two-dimensional (2D)-MRI scans.

	**4D-CT**	**4D-MRI**	**2D-MRI**
Sequence type [-]	Helical	Fast spin echo	Fast spin echo
Orientation [-]	Transversal	Coronal	Transversal
Voxel size [mm^3][a]	0.98–1.37×0.98–1.37×3	1.9×4–6.5×1.9	0.5×0.5×3.5
Field of view [mm^3][a]	500–700×500–700×279–426	457×208–338×350	500×500×140
Number of slices [-]	93–142	52	40
Scan duration [min:s]	1:11–1:48	4:01	4:51–8:15
Gantry rotation [s]	0.44	NA	NA
Detector collimation [mm]	16×1.5		
Pitch factor [-]	0.048–0.089		
Shot mode [-]	NA	Single shot	Multi shot
Sampling [-]		Cartesian	PROPELLER
Contrast [-]		T_2-weighted	T_2-weighted
TE/TR [ms]		64/8042	63/>1800
In-plane parallel imaging factor [-]		2.5	2.6
Echo train length [-]		57	43
Partial Fourier factor [-]		0.625	1
SMS[b] factor [-]		2	1
RNAV[c] length [mm]		NA	100

a) Orientation is LR×AP×CC. b) Simultaneous multi-slice. c) Respiratory navigator.

S2. Deformable image registration

Standard definition mid-position (MidP) computed tomography (CT) images were derived from four-dimensional CT data with a clinically used optical flow deformable image registration algorithm, which is an intensity-based method. This algorithm used a linear (i.e., L_1) data fidelity term instead of the quadratic term in the algorithm proposed by Horn and Schunck, which quantifies the difference between the fixed and transformed moving image, and the L_2-based regularization parameter (spatial smoothness of deformation vector fields (DVFs)) was set to 0.3. The CT data were preprocessed by performing histogram equalization. The algorithm was run twice to obtain the forward and backward DVFs between the reference image and the respiratory phases.

For the MidP magnetic resonance imaging (MRI) registrations, the Advanced Medical Imaging Registration Engine (ADMIRE) adaptive auto algorithm research version 3.47 (Elekta AB, Stockholm, Sweden) was used, which is an intensity-based and feature-constrained method. The latter makes it robust against differences in images intensities, as present in the MRI data from the two different sequences. Although the two MRI scans differed in acquisition orientation and field of view (FOV) for the high-definition (HD)-MidP MRI approach, their FOVs could be aligned using the DICOM reference coordinate system without the need for image registration. The calculation of DVFs by ADMIRE is constrained such that the concatenated forward DVF and backward DVF equal zero. As a result, the algorithm was run only once to obtain both the forward and backward DVFs.

Image quality assessment

Table S4: The criteria of the 4-point Likert scale used to quantify the image quality.

Score	Tumor and organ at risk distinctiveness
**1**	**Very poor distinctiveness**
	Delineations rely on anatomical landmarks and take significant effort
**2**	**Poor distinctiveness**
	Delineations have uncertainties and take moderate effort
**3**	**Good distinctiveness**
	Delineations can be made with little effort and moderate precision
**4**	**Excellent distinctiveness**
	Delineations can be made with minimum effort and a high level of precision

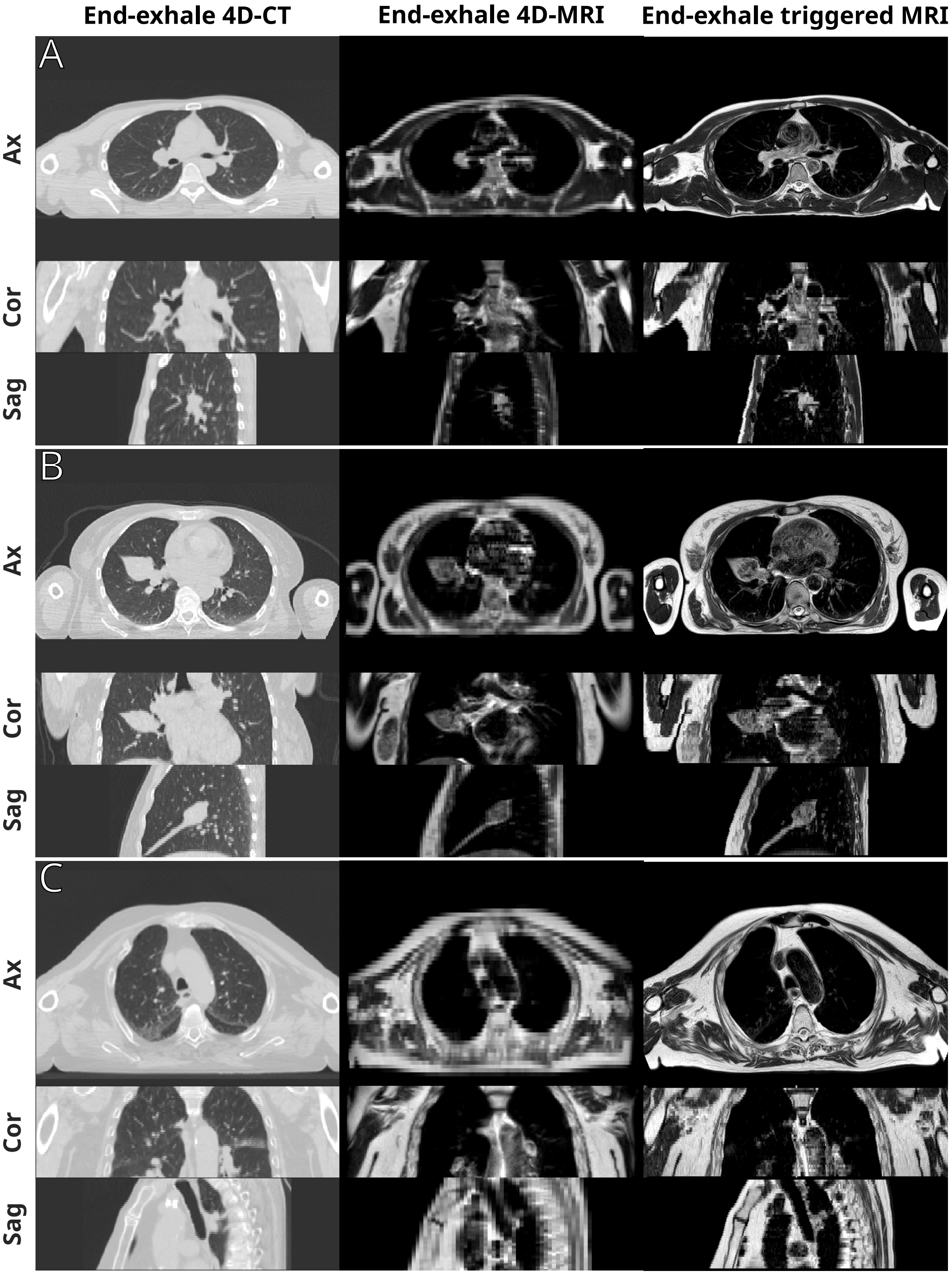

Figure S1: Examples of end-exhale images for the same patients shown in Figure 2 from a four-dimensional computed tomography (4D-CT), 4D- magnetic resonance imaging (MRI), and an end-exhale respiratory-triggered MRI acquisition. The axial (Ax), coronal (Cor), and sagittal (Sag) planes intersecting the tumor location are shown. The CT images are visualized using lung windowing (W:1700, L:−500), whereas MR images have identical windowing (W:1860, L:1070). The (axial) CT data had a slice thickness of 3 mm, the coronal 4D-MRI acquisitions had slice thickness values of 4.0 mm (P2, A), 4.5 mm (P10, B), and 6.0 mm (P12, C), and the axial respiratory-triggered acquisition had a slice thickness of 3.5 mm.

Gross tumor volume

Table S5: Summary of the gross tumor volume (GTV) per individual reader ("R") and the consensus contour based on the contours where at least two readers agreed. Values are summarized for the standard-definition mid-position (SD-MidP) computed tomography (CT), SD-MidP magnetic resonance imaging (MRI), and high-definition (HD)-MidP MRI. Note that Patient 6 had two targets. Furthermore, note the missing values ("NA") for the SD-MidP MRI of P5 and P6b, and for the SD-MidP CT and SD-MidP MRI of P17 by Reader #1.

	SD-MidP CT				SD-MidP MRI				HD-MidP MRI			
Patient	R1 [cc]	R2 [cc]	R3 [cc]	Consensus [cc]	R1 [cc]	R2 [cc]	R3 [cc]	Consensus [cc]	R1 [cc]	R2 [cc]	R3 [cc]	Consensus [cc]
1	1.3	1.0	1.2	1.2	0.7	0.4	0.6	0.5	0.7	0.9	0.7	0.7
2	7.9	3.9	7.0	6.2	5.3	2.8	6.8	5.1	5.4	1.7	9.6	5.2
3	19.1	10.5	12.0	12.0	14.1	6.7	17.5	13.3	14.4	5.8	13.9	12.3
4	3.8	3.7	4.3	3.8	6.0	3.1	6.6	5.3	4.3	3.7	5.7	4.5
5	7.4	9.3	11.1	8.8	NA	NA	NA	NA	3.8	4.0	6.9	4.2
6a	6.7	5.6	5.3	5.8	5.5	4.1	6.1	5.1	3.6	3.6	5.8	4.2
6b	1.3	0.8	1.3	1.1	NA	NA	NA	NA	1.4	0.9	1.4	1.2
7	1.5	0.9	1.5	1.4	1.2	0.9	1.1	0.9	0.8	0.7	1.1	0.8
8	3.3	2.2	5.4	4.2	3.2	2.0	5.3	2.9	3.0	0.5	4.5	2.7
9	21.9	37.3	41.3	33.6	19.8	26.3	38.0	27.3	15.1	28.7	37.7	27.1
10	20.9	8.5	24.3	17.2	16.7	9.3	19.1	14.8	14.6	8.4	23.9	14.6
11	1.2	1.1	1.8	1.3	1.5	0.5	1.3	1.0	1.2	1.0	1.7	1.2
12	19.9	8.3	9.8	12.1	5.3	1.9	3.6	3.0	3.2	5.9	5.0	4.0
13	3.7	2.6	3.4	3.2	2.8	1.6	2.3	2.3	3.3	1.4	3.2	3.0
14	13.6	15.5	14.1	13.8	15.9	10.1	17.3	14.7	10.9	10.2	12.6	11.4
15	17.7	11.9	20.3	16.0	17.2	6.8	16.4	14.5	12.1	7.5	15.0	11.3
16	4.3	3.6	6.1	4.6	7.7	3.5	8.5	6.8	5.2	2.3	5.2	4.6
17	NA	3.5	3.5	2.2	NA	2.0	4.5	1.9	3.7	1.5	3.5	3.2
Mean	9.1	7.2	9.7	8.3	8.2	5.1	9.7	7.5	5.9	4.9	8.7	6.5
Std	7.6	8.4	10.0	8.0	6.4	6.2	9.5	7.2	4.9	6.4	9.1	6.5

Dice similarity coefficient

Table S6: Summary of the Dice similarity coefficient (DSC) scores between gross tumor volume contours of three readers ("R") and the consensus contour based on the contours where at least two readers agreed. Values are summarized for the standard-definition mid-position (SD-MidP) computed tomography (CT), SD-MidP magnetic resonance imaging (MRI), and high-definition (HD)-MidP MRI. Note that Patient 6 had two targets. Furthermore, note the missing values ("NA") for the SD-MidP MRI of P5 and P6b, and for the SD-MidP CT and SD-MidP MRI of P17 by Reader #1.

	SD-MidP CT			SD-MidP MRI			HD-MidP MRI		
Patient	R1 [-]	R2 [-]	R3 [-]	R1 [-]	R2 [-]	R3 [-]	R1 [-]	R2 [-]	R3 [-]
1	0.92	0.65	0.96	0.86	0.76	0.84	0.91	0.56	0.91
2	0.88	0.75	0.90	0.96	0.72	0.84	0.98	0.50	0.71
3	0.73	0.89	0.85	0.96	0.66	0.86	0.92	0.63	0.93
4	0.94	0.81	0.88	0.91	0.69	0.88	0.95	0.81	0.86
5	0.71	0.71	0.89	NA	NA	NA	0.82	0.86	0.76
6a	0.91	0.80	0.89	0.90	0.83	0.90	0.88	0.87	0.82
6b	0.84	0.72	0.83	NA	NA	NA	0.92	0.78	0.88
7	0.93	0.73	0.96	0.81	0.67	0.82	0.93	0.82	0.70
8	0.78	0.58	0.88	0.78	0.80	0.68	0.95	0.33	0.75
9	0.79	0.91	0.89	0.82	0.91	0.84	0.71	0.93	0.84
10	0.87	0.64	0.81	0.92	0.77	0.85	0.95	0.71	0.76
11	0.96	0.87	0.81	0.83	0.61	0.89	0.96	0.87	0.85
12	0.75	0.78	0.80	0.71	0.35	0.87	0.85	0.69	0.85
13	0.92	0.85	0.96	0.88	0.81	0.98	0.94	0.65	0.95
14	0.92	0.86	0.96	0.90	0.79	0.90	0.95	0.86	0.94
15	0.92	0.83	0.87	0.91	0.64	0.93	0.95	0.77	0.84
16	0.90	0.66	0.83	0.91	0.67	0.87	0.93	0.67	0.93
17	NA	0.78	0.78	NA	0.97	0.60	0.92	0.63	0.95
Mean	0.86	0.77	0.88	0.87	0.73	0.85	0.91	0.72	0.85
Std	0.08	0.09	0.06	0.07	0.14	0.09	0.06	0.15	0.08

Mean distance to agreement

Table S7: Summary of the mean distance to agreement (DTA) between gross tumor volume contours of three readers ("R") and the consensus contour based on the contours where at least two readers agreed. Values are summarized for the standard-definition mid-position (SD-MidP) computed tomography (CT), SD-MidP magnetic resonance imaging (MRI), and high-definition (HD)-MidP MRI. Note that Patient 6 had two targets. Furthermore, note the missing values ("NA") for the SD-MidP MRI of P5 and P6b, and for the SD-MidP CT and SD-MidP MRI of P17 by Reader #1.

	SD-MidP CT			SD-MidP MRI			HD-MidP MRI		
Patient	R1 [mm]	R2 [mm]	R3 [mm]	R1 [mm]	R2 [mm]	R3 [mm]	R1 [mm]	R2 [mm]	R3 [mm]
1	0.3	1.1	0.1	0.4	0.7	0.4	0.3	1.4	0.3
2	0.7	1.4	0.6	0.2	1.4	0.9	0.1	2.9	1.9
3	2.3	0.6	1.0	0.3	2.1	1.0	0.5	2.4	0.4
4	0.3	0.9	0.5	0.5	1.4	0.6	0.2	0.8	0.6
5	1.6	2.3	0.7	NA	NA	NA	0.8	0.7	1.5
6a	0.5	1.1	0.6	0.5	0.8	0.5	0.5	0.5	0.8
6b	0.5	1.1	0.6	NA	NA	NA	0.3	0.7	0.4
7	0.2	0.9	0.2	0.6	1.1	0.6	0.2	0.5	0.9
8	1.2	2.0	0.6	1.0	0.9	1.7	0.2	3.5	1.3
9	2.4	0.7	0.9	1.8	0.7	1.8	3.0	0.6	1.5
10	0.9	2.2	1.4	0.6	1.3	1.1	0.3	1.6	2.2
11	0.1	0.4	0.7	0.6	1.3	0.4	0.1	0.4	0.5
12	1.8	1.5	1.4	1.6	3.8	0.5	0.7	1.8	0.7
13	0.4	0.6	0.2	0.5	0.8	0.1	0.3	1.4	0.2
14	0.6	1.1	0.3	0.7	1.4	0.7	0.3	0.8	0.4
15	0.6	1.2	1.1	0.6	2.4	0.4	0.3	1.4	1.1
16	0.4	1.8	0.8	0.4	1.7	0.7	0.3	1.6	0.3
17	NA	1.3	1.0	NA	0.1	2.0	0.3	1.4	0.2
Mean	0.9	1.2	0.7	0.7	1.4	0.8	0.5	1.3	0.8
Std	0.7	0.5	0.4	0.4	0.8	0.5	0.6	0.8	0.6

S3. Treatment margins and planning target volumes

The standard deviation of the deformation vector fields (DVFs) that concatenated the mid-position with the respiratory phases was used to calculate patient-specific anisotropic gross tumor volume (GTV)-planning target volume (PTV) margins ($\text{Margin}_{\text{auto}}$). This calculation employed the nonlinear van Herk margin recipe:

$$\text{Margin} = 2.5\Sigma + 0.67\sqrt{\sigma_p^2 + \sigma^2 + \sigma_b^2} - 0.67\sigma_p. \tag{1}$$

This formula included the residual systematic (Σ) and random (σ) errors, the beam penumbra (σ_p) that equals 6.4 mm in lung tissue obtained from Sonke et al. (2009, Int. J. Radiat. Oncol. Biol. Phys.) and the random error as a result of breathing motion (σ_b). The values for (Σ) and (σ) were adopted from Ligtenberg et al. (2022, Phys. Imaging Radiat. Oncol.) to obtain margins for a 90% population coverage with the 75% isoline: Σ = 1.41 mm and σ = 3.00 mm for the cranial-caudal and anterior-posterior directions, and Σ = 1.12 mm and σ = 2.45 mm for the left-right direction. For comparison, the standard deviation of the manual center of mass (COM)-based translations was used to determine GTV-PTV margins ($\text{Margin}_{\text{man}}$). Based on the margins, PTVs were determined for mid-position (MidP)-based treatment plans.

Figure S2 shows the differences in margins obtained with Equation 1 using manual COM-based translations ($\text{Margin}_{\text{man}}$) and using the DVFs ($\text{Margin}_{\text{auto}}$). Absolute differences between margins were within 0.6 mm, which is smaller than the recommended 2 mm dose calculation grid size by AAPM TG-101. When margins were rounded to the nearest millimeter, which is the clinically used precision, all differences between $\text{Margin}_{\text{man}}$ and $\text{Margin}_{\text{auto}}$ were zero for the MidP magnetic resonance imaging images. Table S8 summarizes the margins based on the DVFs and the resulting PTVs. The uncertainty from the DIR could also be incorporated into the margin recipe. In this study, both the systematic (i.e., anatomical accuracy) and random (i.e., DIR precision) errors had median values of approximately 1 mm and were predominantly within 2 mm. When incorporated simultaneously into the residual systematic and random errors of Equation 1 for all directions, these values led to a generally isotropic increase of 1 mm or 3 mm in the GTV–PTV expansion, respectively. Table S9 summarizes the changes in Σ and σ and their effect on the margin calculation when DIR uncertainty is taken into account.

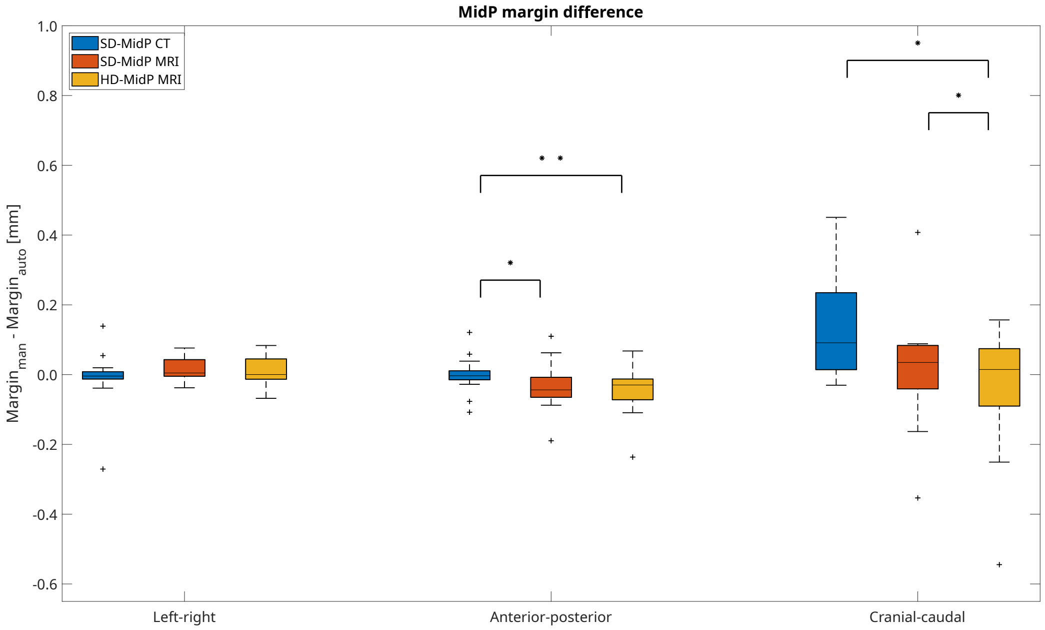

Figure S2: The difference in mid-position (MidP) margins derived using manual center of mass-based translations ($\text{Margin}_{\text{man}}$) and deformation vector fields from image registration ($\text{Margin}_{\text{auto}}$) in three orthogonal directions. Significant differences are marked and indicate p-values below 0.0166 (*) and 0.003 (**). Data outside the whiskers (1.5×interquartile range) were marked as outliers (+).

Table S8: Summary of the consensus gross tumor volume (GTV) and the resulting planning target volume (PTV) based on automatically derived margins for the left-right (M_{LR}), anterior-posterior (M_{AP}), and cranial-caudal (M_{CC}) directions for the mid-position (MidP) concept. Values are summarized for standard-definition (SD)-MidP computed tomography (CT), SD-MidP magnetic resonance imaging (MRI), and high-definition (HD)-MidP MRI. Note that Patient 6 had two targets. Furthermore, note the missing values ("NA") for the SD-MidP MRI of P5 and P6b.

	SD-MidP CT					SD-MidP MRI					HD-MidP MRI				
Patient	GTV [cc]	M_{LR} [mm]	M_{AP} [mm]	M_{CC} [mm]	PTV [cc]	GTV [cc]	M_{LR} [mm]	M_{AP} [mm]	M_{CC} [mm]	PTV [cc]	GTV [cc]	M_{LR} [mm]	M_{AP} [mm]	M_{CC} [mm]	PTV [cc]
1	1.2	3.0	4.0	4.0	4.5	0.5	3.0	4.0	4.0	2.7	0.7	3.0	4.0	4.0	3.4
2	6.2	3.0	4.0	4.0	14.5	5.1	3.0	4.0	4.0	12.8	5.2	3.0	4.0	4.0	13.1
3	12.0	3.0	4.0	4.0	25.4	13.3	3.0	4.0	4.0	27.7	12.3	3.0	4.0	4.0	26.3
4	3.8	3.0	4.0	4.0	10.9	5.3	3.0	4.0	4.0	13.8	4.5	3.0	4.0	4.0	12.4
5	8.8	3.0	4.0	4.0	20.7	NA	NA	NA	NA	NA	4.2	3.0	4.0	4.0	11.8
6a	5.8	3.0	4.0	4.0	14.4	5.1	3.0	4.0	4.0	13.3	4.2	3.0	4.0	4.0	12.2
6b	1.1	3.0	4.0	4.0	4.3	NA	NA	NA	NA	NA	1.2	3.0	4.0	4.0	4.7
7	1.4	3.0	4.0	5.0	5.6	0.9	3.0	4.0	4.0	4.0	0.8	3.0	4.0	4.0	3.6
8	4.2	3.0	4.0	5.0	12.6	2.9	3.0	4.0	5.0	9.8	2.7	3.0	4.0	5.0	9.4
9	33.6	3.0	4.0	4.0	62.2	27.3	3.0	4.0	4.0	50.9	27.1	3.0	4.0	4.0	51.8
10	17.2	3.0	4.0	4.0	35.9	14.8	3.0	4.0	4.0	30.2	14.6	3.0	4.0	4.0	31.2
11	1.3	3.0	4.0	4.0	4.8	1.0	3.0	4.0	4.0	4.1	1.2	3.0	4.0	4.0	4.7
12	12.1	3.0	4.0	4.0	26.0	3.0	3.0	4.0	4.0	8.7	4.0	3.0	4.0	4.0	12.2
13	3.2	3.0	4.0	4.0	8.9	2.3	3.0	4.0	5.0	7.9	3.0	3.0	4.0	5.0	9.4
14	13.8	3.0	4.0	4.0	27.4	14.7	3.0	4.0	5.0	32.5	11.4	3.0	4.0	5.0	26.6
15	16.0	3.0	4.0	4.0	32.3	14.5	3.0	4.0	4.0	31.2	11.3	3.0	4.0	4.0	24.5
16	4.6	3.0	4.0	4.0	13.9	6.8	3.0	4.0	4.0	18.6	4.6	3.0	4.0	4.0	13.7
17	2.2	3.0	4.0	5.0	7.1	1.9	3.0	4.0	5.0	6.6	3.2	3.0	4.0	5.0	9.7
Mean	8.3				18.4	7.5				17.2	6.5				15.6
Std	8.0				14.4	7.2				13.2	6.5				11.9

Table S9: Effect of incorporating deformable image registration (DIR) uncertainty on systematic (Σ) and random (σ) errors. These values were used to derive gross tumor volume (GTV) to planning target volume (PTV) margins using the van Herk margin recipe described in Section S3, with values in parentheses denoting the percentage of patient sets assigned to each margin. Note that Σ and σ values excluding DIR uncertainty were adopted from Ligtenberg et al. (2022, Phys. Imaging Radiat. Oncol.), and the corresponding margins summarize those presented in Table S8.

	DIR uncertainty applied to both Σ and σ simultaneously [mm]		
Direction	0	1	2
**Left-right**			
Σ [mm]	1.12	1.50	2.29
σ [mm]	2.45	2.65	3.16
GTV-PTV margin [mm]	3.0 (100%)	4.0 (100%)	6.0 (98%)<br>7.0 (2%)
**Cranial-caudal/Anterior-posterior**			
Σ [mm]	1.41	1.73	2.45
σ [mm]	3.00	3.16	3.61
GTV-PTV margin [mm]	4.0 (89%)<br>5.0 (11%)	5.0 (92%)<br>6.0 (8%)	7.0 (94%)<br>8.0 (6%)

S4. Maximum intensity projection and average intensity projection images

Maximum intensity projection (MIP) and average intensity projection (AIP) computed tomography (CT) images are commonly used in lung cancer radiotherapy to assess tumor mobility and position probability information, and the MIP can be used to derive the internal target volume (ITV) without contouring the individual four-dimensional (4D)-CT phases. This potentially translates to 4D magnetic resonance imaging (MRI), and therefore MIP and AIP MR images were derived from the 4D-MR image sets and visually compared to the mid-position (MidP) MR images. Figure S3 shows examples of MIP and AIP MR images alongside the derived MidP MR images for two patients. The MIP MR images indicate the ITV similarly as clinically used MIP CT images, which is more pronounced for larger motion amplitudes (example B). The tumor mobility and position probability information encoded in the MIP and AIP MR images, which are obtained without deformable image registration, can be used to validate automatically calculated margins.

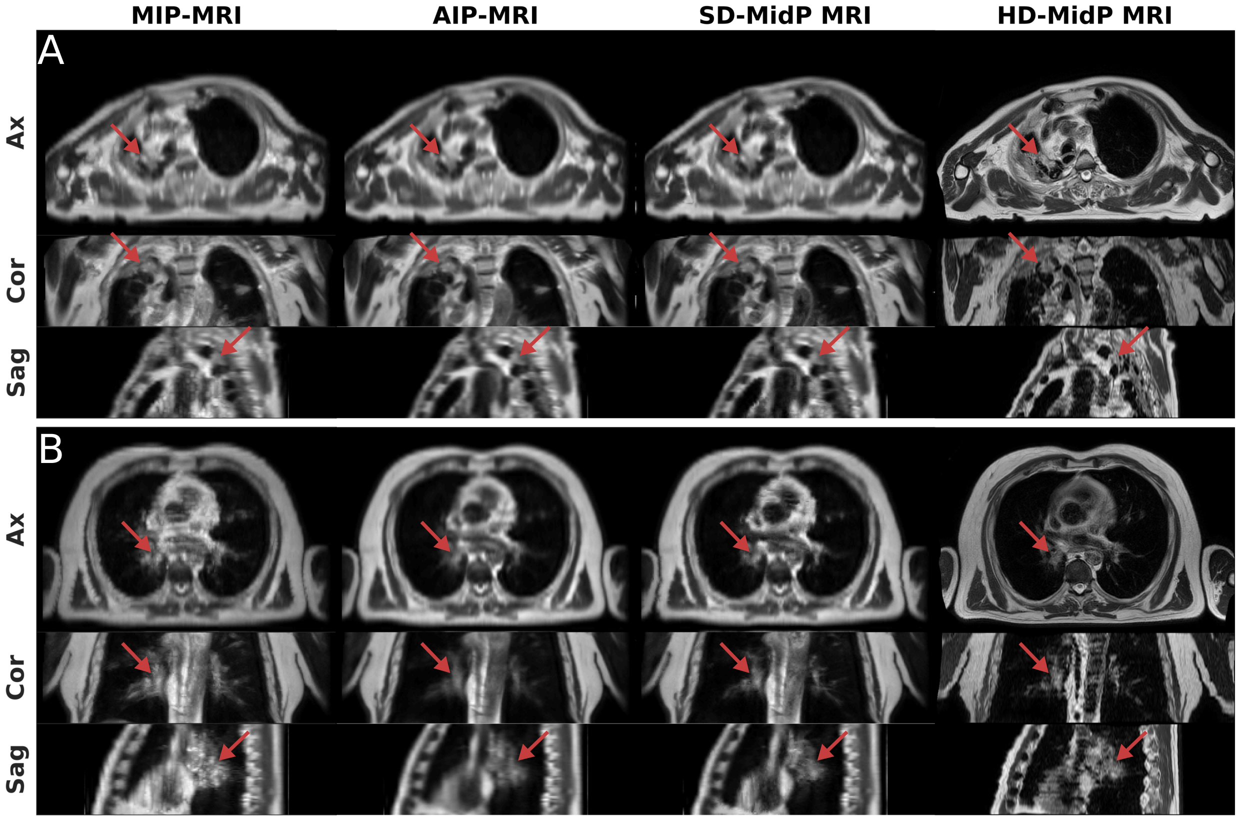

Figure S3: Four different anatomical projections of magnetic resonance imaging (MRI) data for P4 (A) with low tumor mobility (2 mm) and P8 (B) with high tumor mobility (16 mm). Axial (Ax), coronal (Cor) and sagittal (Sag) representations through the tumor location (indicated with the red arrow) are shown. The maximum intensity projection (MIP), average intensity projection (AIP), standard-definition mid-position (SD-MidP), and high-definition (HD)-MidP MRI reconstructions are shown.
